# Combined Immunodeficiency With Late-Onset Progressive Hypogammaglobulinemia and Normal B Cell Count in a Patient With RAG2 Deficiency

**DOI:** 10.3389/fped.2019.00122

**Published:** 2019-04-16

**Authors:** Mayra B. Dorna, Pamela F. A. Barbosa, Andréia Rangel-Santos, Krisztian Csomos, Boglarka Ujhazi, Joseph F. Dasso, Daniel Thwaites, Joan Boyes, Sinisa Savic, Jolan E. Walter

**Affiliations:** ^1^Department of Pediatrics, Faculdade de Medicina da Universidade de São Paulo, São Paulo, Brazil; ^2^Laboratory of Medical Investigation (LIM 36), Department of Pediatrics, Faculdade de Medicina da Universidade de São Paulo, Hospital das Clínicas, São Paulo, Brazil; ^3^Division of Allergy and Immunology, Department of Pediatrics, Morsani College of Medicine, University of South Florida, Tampa, FL, United States; ^4^Department of Biology, University of Tampa, Tampa, FL, United States; ^5^School of Molecular and Cellular Biology, University of Leeds, Leeds, United Kingdom; ^6^Department of Clinical Immunology and Allergy, Leeds Institute of Rheumatic and Musculoskeletal Medicine, St. James's University Hospital, Leeds, United Kingdom; ^7^Johns Hopkins All Children's Hospital, St. Petersburg, FL, United States; ^8^Massachusetts General Hospital, Boston, MA, United States

**Keywords:** RAG deficiency, RAG2, combined immunodeficiency, primary immunodeficiency, hypomorphic variant, compound heterozygous variant

## Abstract

Proteins expressed by recombination activating genes 1 and 2 (RAG1/2) are essential in the process of V(D)J recombination that leads to generation of the T and B cell repertoires. Clinical and immunological phenotypes of patients with RAG deficiencies correlate well to the degree of impaired RAG activity and this has been expanding to variants of combined immunodeficiency (CID) or even milder antibody deficiency syndromes. Pathogenic variants that severely impair recombinase activity of RAG1/2 determine a severe combined immunodeficiency (SCID) phenotype, whereas hypomorphic variants result in leaky (partial) SCID and other immunodeficiencies. We report a patient with novel pathogenic compound heterozygous *RAG2* variants that result in a CID phenotype with two distinctive characteristics: late-onset progressive hypogammaglobulinemia and highly elevated B cell count. In addition, the patient had early onset of infections, T cell lymphopenia and expansion of lymphocytes after exposure to herpes family viruses. This case highlights the importance of considering pathogenic RAG variants among patients with preserved B cell count and CID phenotype.

## Introduction

RAG1 and RAG2 proteins combine to form a heterotetrameric complex which acts as an endonuclease. The RAG recombinase complex performs the essential first step in recombining variable (V), diversity (D), and joining (J) segments of antigen receptor genes that generate the diversity of T and B cell receptors (TCR, BCR) (1). TCR and BCR are important not only for the function of antigen recognition by T and B cells, but also for their development and survival. Variants in *RAG1* and *RAG2* are associated with many different immunodeficiencies including T^−^B^−^NK^+^ SCID, Omenn syndrome (OS), leaky SCID (LS) with γδ T cell expansion, and combined immunodeficiency with granulomas and/or autoimmunity (CID-G/AI) (2–5). Increasing access to genetic studies of immunodeficient patients is expanding the spectrum of phenotypes attributed to RAG deficiency. More recently, hypomorphic *RAG* variants were identified in patients with CD4+ T cell lymphopenia, and phenotypes resembling common variable immunodeficiency (CVID) and specific anti-polysaccharide antibody deficiency affecting all ages (6–10). In this report, we present a patient with CID associated with novel compound heterozygous *RAG2* variants. The clinical and immune phenotype includes early onset of infections, T cell lymphopenia, normal B cell count, late onset progressive hypogammaglobulinemia, and evolving expansion of T and B lymphocytes with exposure to herpes family viruses.

### Case Presentation

A 14-year-old female with chronic rhinosinusitis and lung disease with bronchiectasis was referred for immunologic investigation in São Paulo, Brazil. She had a history of chronic cough with recurrent wheezing since birth with prolonged use of antimicrobials for lower and upper respiratory tract infections, oral candidiasis and stomatitis. She had one episode of pneumonia and she was never hospitalized. She is an offspring of non-consanguineous parents. One of her sisters died with leukemia at the age of 9 months, and her mother experienced recurrent pneumonias and otitis media in childhood.

At 8.5 years of age, pulmonary symptoms worsened, bronchiectasis was detected on computed tomography and pulmonary function assessment showed mild obstructive lung disease. Cystic fibrosis and ciliary dyskinesia were excluded. She was treated with inhaled corticosteroids, azithromycin and chest physical therapy for 2 years with poor clinical response.

Immune evaluation was performed at several time points in the period of 8.5–14.3 years of age (Tables 1, 2). Total lymphocyte count was grossly preserved. Immunoglobulin (Ig) levels were variable with low IgA, low to normal IgG and low to high IgM initially. By 10 years of age, laboratory evaluation showed low levels of all Ig isotypes and low CD4+ and CD8+ T cells with low fraction of CD45RA+ naïve cells and skewing to activated memory T cell phenotype. Lymphocyte proliferation was normal with mitogens but impaired with antigen stimulation (Table 1). As Ig levels decreased, treatment with intravenous Ig (IVIG) was initiated at 11 years of age (Table 2). There was no evidence of protein loss. The B cell developmental subsets were significantly skewed with a marked decline in the switched memory B cell compartment (Table 3). B cell dysfunction is also reflected by decreased total IgG, IgM, and IgA levels with increased age (Table 2). Anti-thyroglobulin and anti-thyroperoxidase antibodies were persistently positive with normal thyroid function.

**Table 1:** Lymphocytes subsets profile (count/microliter) and lymphocyte proliferation to mitogens and antigens over 4 years [stimulation index (SI^*^)].

**Lymphocyte subset absolute (count/microliter)**		**Age (years)**					**Normal value (10–15 years)**
		**10.5**	**12**	**12.6**	**13.9**	**14.3**	
Lymphocytes		1,110 (L)	1,640	2,140	3,650	2,880	>1,300
CD3 T cells		617 (L)	509 (L)	706 (L)	1,619	1,042	>1,000
CD4 T cells		285 (L)	285 (L)	302 (L)	680	367 (L)	>530
CD8 T cells		342 (L)	224 (L)	360 (L)	980	662	>350
CD19 B cells		504		1,246	1,681	1,339	>110
CD56 NK cells		380		156	201	468	>70
CD4+CD45RA+ T cells			53 (10.4%) (L)				>37%
Lymphocyte proliferation to mitogens and antigens (SI)^*^	PHA		15.93			33.3	>18
	PWM					35.1	>8
	Anti-CD3					63	>15
	TT					0.8 (L)	>3.6
	CMV					0.7 (L)	>3.6
	candida					1.6 (L)	>3.3

*L, low; NK, natural killer; PHA, phytohemagglutinin; PWM, pokeweed mitogen; CD, complementarity-determining region; TT, tetanus toxoid; CMV, cytomegalovirus*.

* *The stimulation index is the ratio of counts per minute of stimulated vs. unstimulated (background) cells*.

**Table 2:** Immunoglobulin (Ig) levels and anti-cytokine antibodies before and after IgG replacement therapy, which was begun at 11 years of age.

**Ig class**	**Age (years)**				
	**8.5**	**9.6**	**10.8**	**11**	**14.1**
IgG (mg/dL)	1,055 (633–1,280)	713 (L) (1,000–1,516)	537 (L) (970–1,710)	454 (L) (970–1,710)	1,118^*^ (639–1,349)
IgM (mg/dL)	225.5 (59–151)	66 (59–151)	32.6 (L) (53–145)	25.9 (L) (53–145)	16.4 (15–188)
IgA (mg/dL)	13.8 (L) (33–220)	<50 (L) (45–234)	<5 (L) (45–234)	<0.1 (L) (69–382)	0.1 (L) (47–259)
IgE (IU/mL)	<4 (<90)	5 (<90)	<0.1 (<200)	<0.1 (<200)	<0.1 (<200)
Anti-cytokine antibodies			Anti-IFN-α Anti-IFN-ω Anti-IL-12		Present (H) Absent Absent

*Normal range of Ig class concentrations in parentheses*.

*L, low; H, high; IFN, interferon; IL, interleukin*.

IFN-α, interferon alpha; IFN-ω, interferon omega; IL-12, interleukin 12

* *On IvIg*.

**Table 3:** B cells subset percentages at 13.9 and 14.3 years of age.

**B cell subsets**	**13.9 (years)**	**14.3 (years)**
CD19 (% of live lymphocytes)	78.4% (H) (14.9%)^*^	56.5% (H) (9.9%)^*^
Naïve (% of CD19+)	70.4% (57.4%)	61.9% (59.9%)
Memory (% of CD19+)	12.9% (L) (33.5%)	19.6% (31.7%)
IgD only (% of CD27+)	20.6% (24.9%)	–
IgM only (% of CD27+)	13.3% (13.3%)	–
IgD IgM positive (marginal zone) (% of CD27+)	59.6% (30.8%)	–
IgD IgM negative (switched) (% of CD27+)	6.5% (L) (31.0%)	–
Double negative (% of CD19+)	14.9% (8.3%)	15.7% (7.4)

* *Values in parentheses belong to a healthy control donor that were measured in the same experiment as that of the patient. H, high; L, low*.

**Figure 1:**
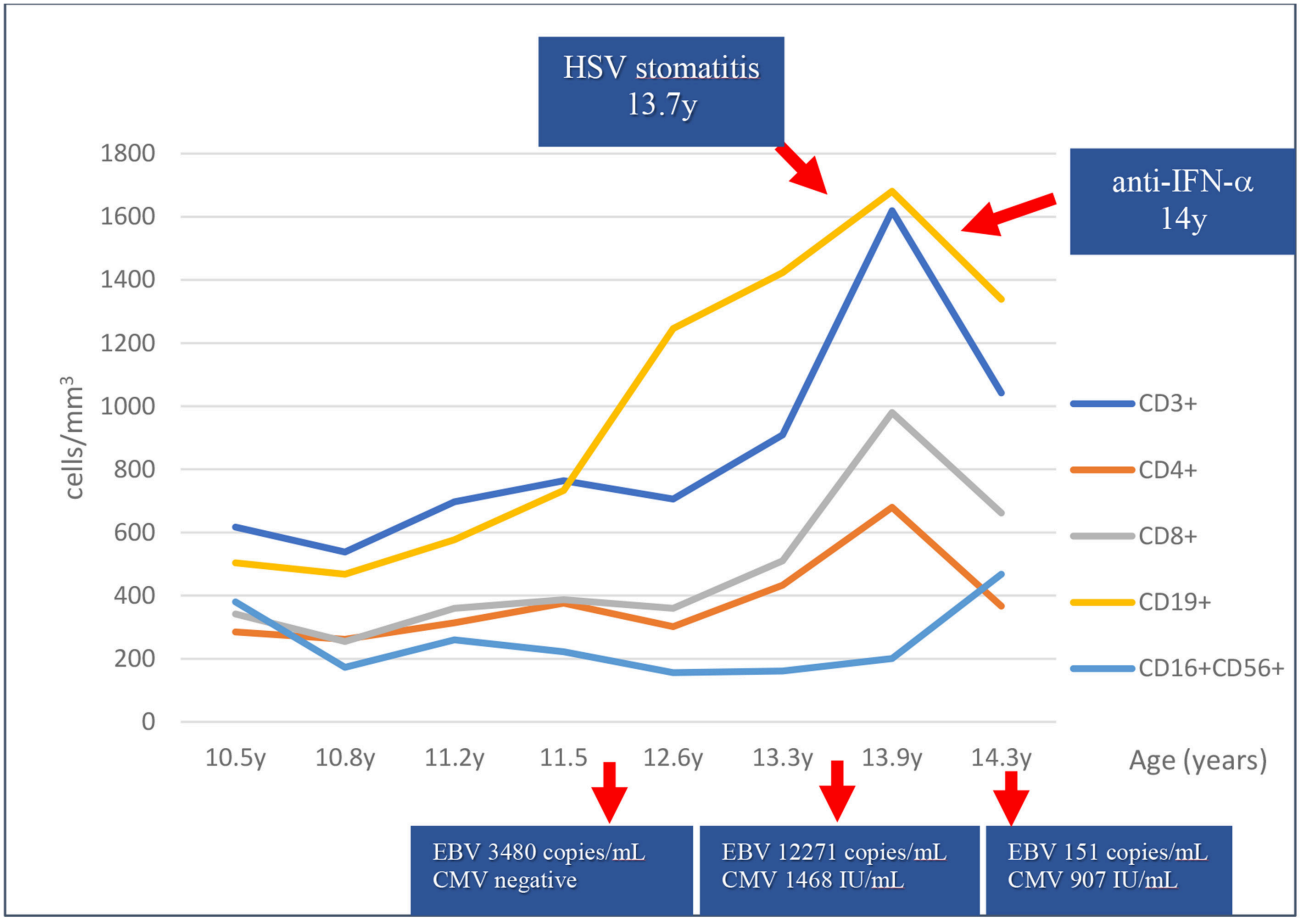
Lymphocyte subset counts and viral copy numbers over 4 years. An increase of T and B cell counts has been observed in the last 2.5 years coincidently with cytomegalovirus (CMV) and Epstein-Barr virus (EBV) viremia, and herpes simplex virus (HSV) stomatitis as shown by polymerase chain reaction. Anti-interferon-alpha antibodies (anti-IFN-α) were present at 14 years of age.

Regarding infectious complications, symptoms of respiratory infections improved on intravenous immunoglobulin G (IVIG) replacement therapy. However, recurrent candidiasis continued to occur as well as episodes of oral ulcers. At 13 years of age, she was hospitalized with bilateral pneumonia and stomatitis with positive polymerase chain reaction (PCR) for herpes simplex virus (HSV) on oral lesion biopsy. She responded well to intravenous antibiotics and acyclovir. Persistently, Epstein-Barr virus (EBV) and intermittently, cytomegalovirus (CMV) viral loads were detected without obvious clinical manifestations of lymphoproliferation or acute viral distress since 11.8 years for EBV and 13.4 years of age for CMV (Figure 1). A progressive increase in CD8+ T cells, B cells (CD19+) and natural killer (NK) cells (CD16+CD56+) was noticed in the same period (Figure 1). Testing at 14 years of age revealed the presence of antibodies targeting interferon-alpha (anti-IFN-α) (Figure 1).

Genetic studies using targeted sequencing of 16 SCID genes identified a heterozygous compound variant in *RAG2* (c.509A > G: p.E170G and c.829insT, p.Y277fs) in the coding regions. Both components of the compound variant were novel. The heterozygous variant was confirmed by Sanger sequencing. The c.509A > G, p.E170G variant was present in the mother. The father was unavailable for study.

Based on prediction analysis and structural modeling, it is expected that the mutated RAG2 allele with Y277fs will not contribute any recombinase activity as truncation of RAG2 further than amino acid 350 leads to a non-functioning protein that is unlikely to form any complex with RAG1 (11). Mutation in the RAG2 E170 is likely important in RAG1/2 dimerization as it contacts an arginine residue in RAG1 (R561). Mutation of RAG1 R561 to histidine has been previously reported in Omenn patients and shown to have reduced DNA binding and cleavage activity, but retains some activity *in vitro* (12). Therefore, we predicted that RAG2 E170G will perform all of the recombinase activity in the patient.

**Figure 2:**
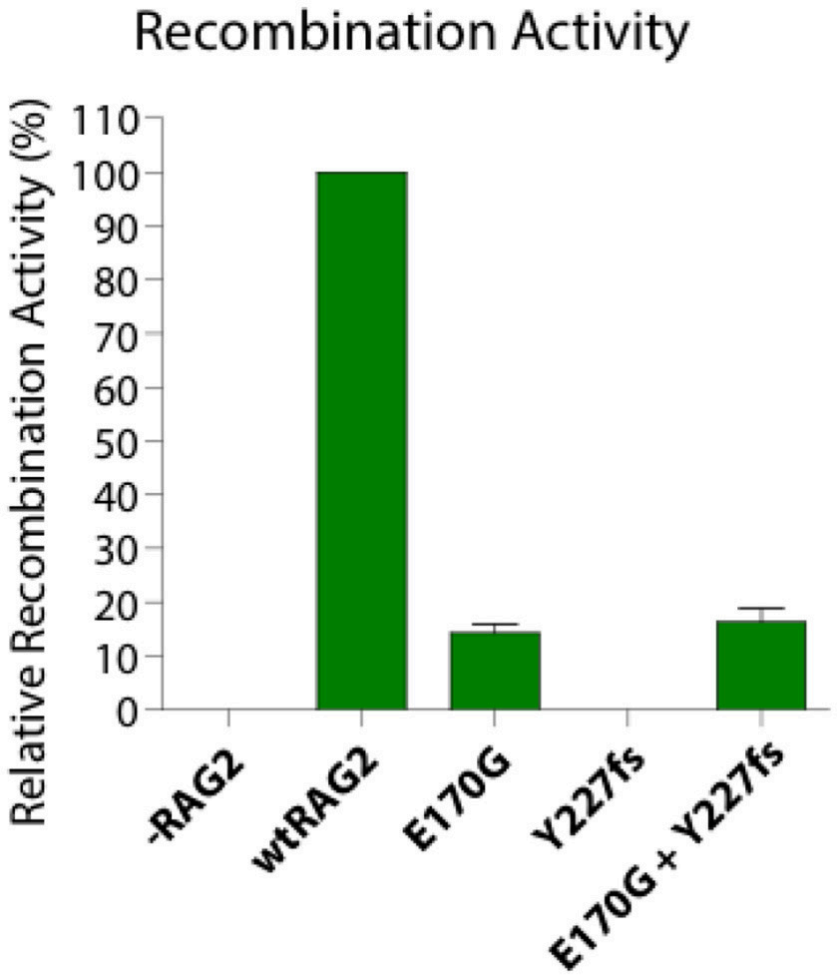
Recombination activity of two heterozygous *RAG2* pathogenic variants (E170G, Y227fs) alone and in combination relative to the wild type allele (wtRAG2). -RAG2 is a negative control.

The relative recombinase activity of the RAG2 variants *in vitro* individually and in a bi-cistronic system was tested using methods previously reported (13). The relative recombinase activity level of the protein expressed by *RAG2* p.E170G variant was 14.2% ± 1.6 standard error of mean (SEM) whereas the p.Y227fs variant had zero activity and their combined activity was 16.2% ± 2.5 SEM in an *in vitro* system (Figure 2). Thus, the RAG2 E170G variant solely contributed to the partial recombinase activity of the patient.

Although considered, the family elected to wait with hematopoietic stem cell transplantation. Regarding donor selection, there is no matched sibling available and we await results of human leukocyte antigen testing to search for a matched unrelated donor.

## Methods

Targeted genes sequencing was performed using genomic DNA extracted from peripheral blood with Qiagen columns (QIAamp DNA Mini Kit; Qiagen, Hilden, Germany) according to the manufacturer's instructions. PCR was carried out by a set of primers designed using www.bioinformatics.nl/primer3plus for all genes (IL2RG:NM_000206, ADA:NM_000022, JAK3:NM_000215, ORAI1:NM_032790, RAG1:NM_000448, RAG2:NM_001243785, GATA2:NM_001145662, CD247:NM_000734, CD3D:NM_000732, CD3E:NM_000733, CD3G:NM_000073, LCK:NM_005356, NHEJ1:NM_024782, IL7R:NM_002185, LIG4:NM_206937, and PNP:NM_000270). The genes were sequenced using targeted sequencing performed with Nextera®XT DNA Library Preparation Kit (Illumina, San Diego, CA, USA) according to the manufacturer's instructions. Variants were identified using Platypus software (Welcome Centre for Human Genetics, Oxford, UK) and annotated using Annovar, an open source software tool. In order to predict the impact of these variants on the protein function, we employed *in silico* prediction tools: PhyloP, SIFT, PolyPhen-2, LRT and VariantTaster. The variants considered important were Sanger sequenced. PCR products were directly sequenced with BigDye Terminators (version 3.1) and analyzed on a Genetic Analyzer (Applied Biosystems. Foster City, CA, USA). B and T cells subset populations were determined by flow cytometry and analyzed using FlowJo software (Treestar, Ashland, OR, USA). Anti-cytokine antibodies were measured by enzyme-linked immunosorbent assay as previously described (4). Recombinase activity level was determined using bi-cistronic *in vitro* method performed in triplicate as described by Thwaites et al. (13).

## Discussion

Impaired RAG function is associated with different clinical and immunological phenotypes and usually presents with variable degrees of B and T cell lymphopenia and antibody deficiency. The reported RAG deficient patient has a CID phenotype with two distinctive characteristics: late-onset progressive hypogammaglobulinemia and normal B cell count. The patient does not meet the criteria for LS or OS as mitogen proliferation are preserved, and has higher relative RAG activity [16% in combined variants (Figure 2)] compared to LS/OS patients. This is consistent with most CID patients having RAG activity ranging from 10 to 60% of wild type RAG activity (12, 14).

Late onset progressive hypogammaglobulinemia has been previously identified in patients with pathogenic RAG variants. Buchbinder et al. (8) reported two patients who not only had decreasing Ig levels but also a later onset of clinical symptoms, both of which contributed to the delay in correct diagnosis. Patients with late onset CID (LOCID) may be misdiagnosed with CVID due to similarities in clinical and immunologic characteristics (8). Nevertheless, patients with LOCID usually present with lower T cell counts and a marked decrease in CD4+ T cells in particular naïve T cells, compared to CVID patients (15). The findings that led to the hypothesis of CID in our patient and motivated the search for the underlying genetic defect were early onset of recurrent bacterial, viral and fungal infections associated with marked reduction of CD4+ and CD45RA naïve T cells with skewing to activated memory phenotype.

Another interesting immunologic finding in this patient is the presence of a normal B cell count. Due to the importance of the recombinase activity of RAG1/2 for B cell development and repertoire diversity, RAG variants are usually associated with marked B cell lymphopenia. However, normal B cell count has been found in other young patients with RAG variants, including patients with CID, ages 2.5 months and 2 years, respectively (16, 17). In both cases, the patients had active herpes virus infections (CMV and varicella specifically). The oldest RAG deficient patient reported with normal B cell count has been recently published by our group [Patient #12 in Lawless et al. (10)]. This 64 year-old female patient with RAG1 compound heterozygous variants (M435V and M1006V) and relative recombinase activity of 28%, had a normal B cell count of 216 (cells/μl) (10% of total lymphocytes). This patient had no history of major infections and had a sole autoimmune manifestation of pernicious anemia. She is currently on Ig replacement therapy with no inflammatory lung complications.

The B cell count may be preserved because of the lymphoproliferation (17). Kuijpers et al. (6) found a disturbed B cell population distribution in a RAG deficient patient with CD4+ T cell lymphopenia; an extensive antigen-independent proliferation of the transitional, naïve and non-switched B cells apparently accounted for the normal B cell count in peripheral blood. Our patient shows similar phenomena in B cell developmental abnormalities.

Expansion of gamma-delta T lymphocytes driven by severe herpes family viral infection have been described in LS patients with RAG variants (18, 19). Interestingly, we noticed that our patient presented a marked increase in both B cell and CD8+ T cells counts and a lesser increase in CD4+ T cells during herpes family virus asymptomatic viremia (CMV and EBV) and stomatitis (HSV) (Table 1, Figure 1). The reduction of EBV and CMV copies in peripheral blood and the resolution of the stomatitis were followed by a reduction in B and T cell counts.

Late onset progressive hypogammaglobulinemia is less common in combined immunodeficiencies that present early in life with profound antibody deficiency. However, key feature of late onset progressive hypogammaglobulinemia occurs among patients with IKAROS deficiency (20). Large studies have not been published on naïve IgG levels of RAG deficient patients in different age groups as most published patients receive Ig replacement therapy early in life. Therefore, it is yet to be determined how large is the subgroup with late onset progressive hypogammaglobulinemia.

Indication of hematopoietic stem cell transplantation (HSCT) are unclear for late onset CID and RAG deficiency. The patient is clinically well on Ig replacement therapy. There is still concern for progressive inflammatory lung disease, or occurrence of autoimmune complication, as reported by our group (10). Only a selected number of adolescent and adults with partial RAG deficiency received HSCT with variable success (21, 22). Allogeneic HSCT is a viable option for our patient; we are discussing with the family whether it is worthwhile given the patient's good response to current treatment vs. the risk of potential inflammatory and/or autoimmune sequela. With recent advances in gene therapy, our patient may be a candidate for this approach as well. The compound heterozygous variant, however, may create another layer of complexity in this process.

In conclusion, we found a novel compound heterozygous *RAG2* variants that results in 16% relative recombinase activity and CID phenotype with normal B cell count but skewed B cells subsets and late-onset hypogammaglobulinemia. RAG deficiency may present with many different phenotypes and should be considered in patients with early-onset infections. This is especially true with T cell lymphopenia and low naïve T cell counts, even with normal B cell counts and late-onset hypogammaglobulinemia as in our case study. The immune phenotype can be modified by herpes viral infection including skewed B cell expansion and generation of anti-cytokine antibodies in temporal association of chronic herpes viral infections as seen in our case and previous reports (4, 17). Ig levels can decrease with time and should be monitored for early intervention with Ig therapy. Identifying the underlying genetic defect can greatly facilitate diagnosis of new phenotypes and enable effective therapy, including HSCT.

## Ethics Statement

This study was performed in accord with the recommendations of the ethics committee of Hospital das Clínicas da Faculdade de Medicina da Universidade de São Paulo. The mother gave written informed consent for the laboratory investigations in this case report and for publication of the study.

## Author Contributions

MD, PB, and JW wrote the manuscript. MD, PB performed clinical and laboratory data collection and analysis. MD followed the patient. AR-S performed genetic studies. KC and BU performed B cell and anti-cytokine antibody studies. JW contributed to the interpretation of genetic data. DT performed and JB and SS gave advice on recombinase activity. JD and JW provided a critical revision of the article, and JW gave final approval of the version to be published.

### Conflict of Interest Statement

The authors declare that the research was conducted in the absence of any commercial or financial relationships that could be construed as a potential conflict of interest.
